# Bone marrow CD8 T cells express high frequency of PD-1 and exhibit reduced anti-leukemia response in newly diagnosed AML patients

**DOI:** 10.1038/s41408-018-0069-4

**Published:** 2018-03-21

**Authors:** Bei Jia, Liru Wang, David F. Claxton, W Christopher Ehmann, Witold B. Rybka, Shin Mineishi, Syed Rizvi, Hiroko Shike, Michael Bayerl, Todd D. Schell, Raymond J. Hohl, Hong Zheng

**Affiliations:** 1 Penn State Cancer Institute, Penn State University College of Medicine, Hershey, PA 17033 USA; 2 Fu Xing Hospital, Capital Medical University, Beijing, 100038 China; 3 Department of Medicine, Division of Hematology-Oncology, UT Southwestern Medical Center, Dallas, TX 75390 USA; 4 Department of Pathology, Penn State University College of Medicine, Hershey, PA 17033 USA; 5 Depatment of Microbiology and Immunology, Penn State University College of Medicine, Hershey, PA 17033 USA

Acute myeloid leukemia (AML) is a devastating blood cancer with 5-year survival of only 25%. Novel effective leukemia therapy is an urgent unmet need. Immunotherapy is a promising strategy for cancer treatment. Targeting inhibitory mechanisms, such as the programmed cell death protein 1 (PD-1) pathway to unleash the patient’s own anti-tumor immune response has achieved major success^1^. Several studies including ours have demonstrated an involvement of PD-1 and other T cell inhibitory pathways in AML progression^2–7^. Strategies of blocking immune suppression for leukemia treatment are attractive due to their relatively simple administration and better tolerability profile. In fact, clinical studies applying anti-PD-1 in AML therapy have been initiated and are currently in early phase trials^8^. AML is a highly heterogeneous disease with multiple steps involved in the treatments. Determining the status of the immune response at each disease stage in individual patients is crucial for decision making of subsequent clinical management. In addition, immune characterization has great potential to identify predictive biomarkers of responsiveness to immunotherapy, thus providing pivotal information to optimize clinical trial design using strategies modulating anti-leukemia immunity for AML treatment.

Due to limited accessibility, the majority of clinical studies in AML have been restricted to evaluation of peripheral blood samples. In contrast, the immune response in bone marrow of AML patients is poorly understood. AML is derived from myeloid hematopoietic progenitors and rapidly grows in bone marrow before mobilizing to peripheral blood in a majority of patients. Therefore the development and progression of AML largely relies on the bone marrow microenvironment^9^. A better understanding of the anti-leukemia immune response within the bone marrow of AML patients is likely to be a key to develop immune-based therapeutic approaches for leukemia. In this study, we investigated the T cell immune response within the bone marrow in AML.

Samples collected from a cohort of 22 patients with newly diagnosed AML were used in this study. Clinical and demographic information are summarized in Supplementary Table 1. Consistent with the heterogeneous nature of AML, there was wide variation in the white blood cell (WBC) count and percentage of blasts in both peripheral blood and bone marrow. The risk stratification based on cytogenetic features was defined by European Leukemia Net (ELN) 2017 recommendations^10^. Two patients (9.1%) were categorized with favorable risk, ten (45.5 %) with intermediate risk, and the other ten patients (45.5 %) were in the adverse risk category. Peripheral blood and bone marrow aspirates were collected from each patient prior to any leukemia treatment.

We first assessed the T cell composition of paired bone marrow and peripheral blood from the same patients. We observed that the frequency of CD3^+^ T cells among lymphocytes is significantly lower in bone marrow than in peripheral blood (Fig. 1a). Percentages of CD4 and CD8 T cell subsets among CD3^+^ T cells were comparable between bone marrow and peripheral blood. While the CD4/CD8 ratio was similar, the frequency of CD4 and CD8 T cell among lymphocytes was significantly lower in bone marrow (Fig. 1a). Our result is in line with reports that in healthy individuals, the bone marrow contains a lower percentage of CD3^+^ T lymphocytes compared with peripheral blood^11^. However, in contrast to the observations that healthy individuals contain comparable frequency of CD8 T cells among lymphocytes within the two anatomical locations^11^, our data demonstrate a diminishment of the CD8 T-cell subset in bone marrow of AML patients, suggesting a suppressed anti-leukemia CD8 activity in the marrow. Our further phenotypic analysis of T cells derived from AML patients showed that the majority of CD8 T cells in bone marrow are effector memory T cells (T_EM_), and the percentage of T_EM_ in bone marrow is significantly higher than that of peripheral blood (Fig. 1b). Previous studies have demonstrated that bone marrow is a unique anatomical site with enrichment of memory T cells in healthy individuals and multiple disease conditions including viral infection, degenerative joint disease, and solid tumors^12,13^. Our finding is consistent with these observations and we similarly conclude that the bone marrow of patients with AML is also a reservoir for memory T cells.

**Fig. 1:**
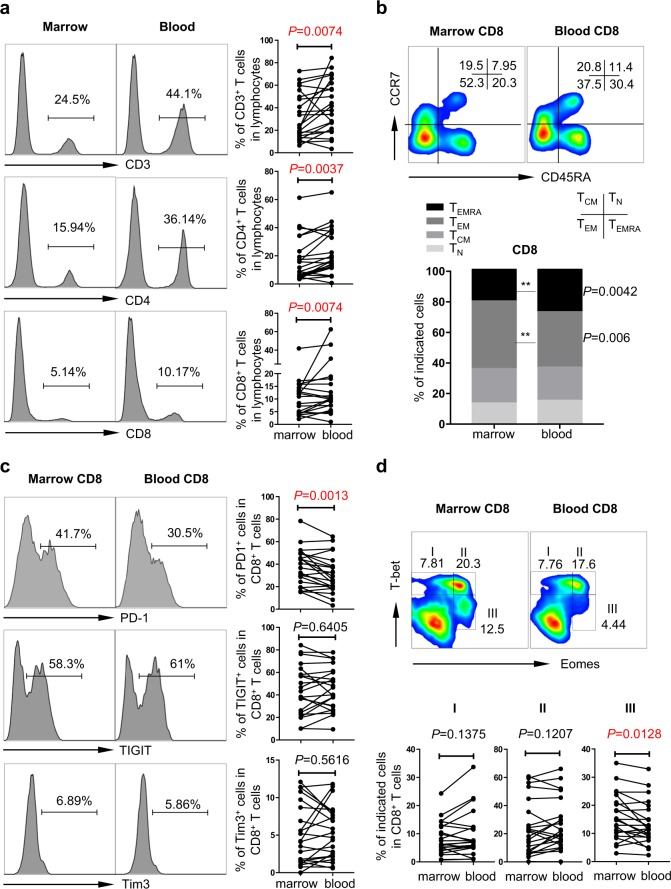
Bone marrow CD8 T cells express high frequency of PD-1 and contains more Eomes^hi^T-bet^int^ cells than peripheral blood in AML. Paired bone marrow and peripheral blood samples were collected from newly diagnose AML patients (*n* = 22). Flow cytometry analysis was performed on peripheral blood mononuclear cells (PBMCs) and bone marrow mononuclear cells (BMMCs). **a** The percentages of CD3^+^, CD4^+^, and CD8^+^ T cell within lymphocytes are shown. Histogram (left) displays the representative flow cytometry data. Panels on right are the summary plot of 22 patients. **b** Distribution of T_N_, T_CM_, T_EM_, and T_EMRA_ among CD8 T cells in bone marrow and peripheral blood was evaluated based on the expression of CD45RA vs. CCR7. Representative flow data (top) and summary plot (bottom) are shown. **c** Flow cytometry analysis of the surface expression of PD-1, TIGIT, and TIM-3 was performed. Data of CD8 is shown. Representative histograms (left) and statistic summary plots (right) display the expression level of indicated inhibitory receptors. **d** Flow cytometry analysis of the intracellular expression of Eomes vs. T-bet among CD8 T cells was performed. Based on the levels of Eomes and T-bet expression, cells are divided into three fractions. The schema of each fraction is shown in representative flow data (top). Panels at the bottom display the summary of expression levels of I, II, and III among CD8 T cells. *P* values were obtained by the paired *t* test and Wilcoxon matched pairs signed rank test

Several studies including ours have demonstrated an involvement of the inhibitory receptors PD-1 and T cell immunoglobulin and mucin domain (TIM-3) in AML progression^2–6^. We also discovered a suppressive effect of T cell immunoglobulin and ITIM domain (TIGIT), a recently identified co-inhibitory receptor, in the CD8 T cell response in AML^7^. To evaluate whether the effect of inhibitory pathways is different between bone marrow and peripheral blood, we assessed the expression of PD-1, TIGIT, and TIM-3 on T cells derived from both sites. We observed a significantly increased frequency of PD-1-expressing CD8 T cells in bone marrow compared with that of peripheral blood (Fig. 1c). There were no significant differences in the proportion of CD8 T cells expressing TIGIT or TIM-3 (Fig. 1c). PD-1 is a key mediator for the development of T cell exhaustion, a state of T cell dysfunction that develops in response to persistent antigen stimulation, including cancer. Our observation that a higher proportion of bone marrow CD8 T cells express PD-1 suggests a more exhausted status of T cells and suppressive environment within bone marrow in AML patients. We further examined the expression of Eomesodermin (Eomes) and T-bet in CD8 T cells and found that the frequency of Eomes^hi^T-bet^int^ CD8 T cells was significantly higher in bone marrow compared with that of peripheral blood (Fig. 1d). Eomes and T-bet are both T box transcription factors that are crucial in regulating T cell function. Recent studies demonstrated, in both a mouse model and human chronic viral infection, that the T-bet^hi^ subset of exhausted T cells retains some proliferative capacity and can be reinvigorated by PD-1 blockade. In contrast, Eomes^hi^ T cells are terminally differentiated and irreversible^14^. Our observation that there are more Eomes^hi^ CD8 T cells in bone marrow indicates that this microenvironment may skew these T cells toward the late stage of exhaustion.

To evaluate the functional status of CD8 T cells in bone marrow. We performed multiple functional studies to assess the cytokine production, proliferative ability, and killing capacity. We found no significant difference in cytokine release (IFN-γ and TNF-α) upon in vitro stimulation with anti-CD3 and anti-CD28 (Fig. 2a, b). In addition, the expression of Ki67 on CD8 T cells was comparable between bone marrow and peripheral blood, demonstrating a similar proliferation (Fig. 2c). Strikingly, intracellular expression of Granzyme B in CD8 T cells from bone marrow was significantly lower compared with that of peripheral blood (Fig. 2d), suggestive of an impaired killing capacity of CD8 T cells in bone marrow. To further dissect the function of leukemia-reactive CD8 T cells in bone marrow vs. that in peripheral blood, we tested CD8 T cells for cytokine release in response to a WT-1 peptide. WT-1 is a well know tumor-associated antigen, in which HLA-A*0201 restricted WT-1_126–134_ is the most studied epitope in AML^15^. We found a significantly lower production of both IFN-γ and TNF-α by CD8 T cells from bone marrow compared with that from peripheral blood (Fig. 2e). Furthermore, when leukemia-reactive CD8 T cells were evaluated (gated on IFN- γ^+^), we observed a significantly higher expression of PD-1 on cells derived from bone marrow compared with that of peripheral blood (Fig. 2f). These important data demonstrate that in patients with AML, bone marrow leukemia-reactive CD8 T cells consist of a higher frequency of PD-1^+^ cells and are functionally deficient compared with that of peripheral blood. This novel finding provides a strong rationale for therapeutic strategies targeting inhibitory mechanisms including PD-1 to enhance the anti-leukemia response in AML patients.

**Fig. 2:**
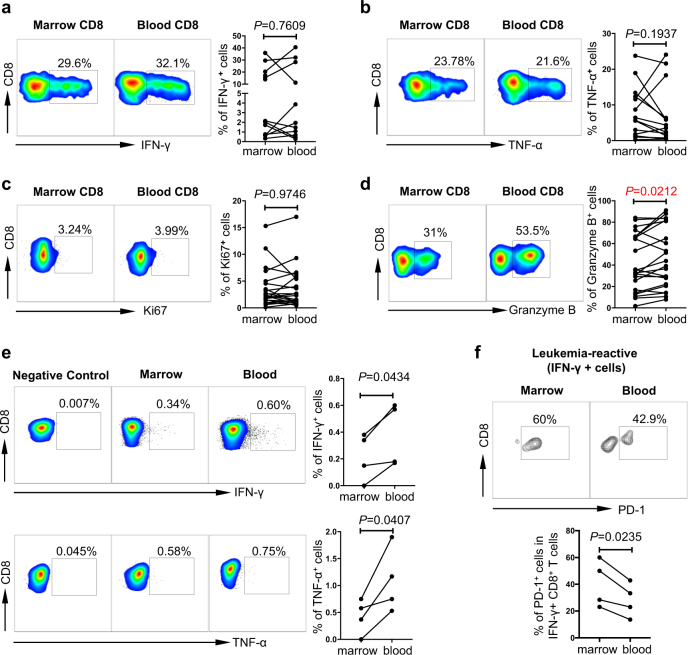
Functional status of total CD8 T cells and leukemia-reactive CD8 T cells in bone marrow vs. peripheral blood of AML. **a**, **b** PBMCs and BBMCs collected from AML patients at initial diagnosis (*n* = 10) were stimulated in vitro with anti-CD3 and anti-CD28 before intracellular staining with IFN-γ and TNF-α. Flow cytometry analysis of the expression of IFN-γ (**a**) and TNF-α (**b**) are shown. Left panels, representative flow data; right panels, summary plots. **c**, **d** Expression of Ki67 (**c**) and Granzyme B (**d**) in CD8 T cells from bone marrow and peripheral blood of AML patients (*n* = 22) was assessed by flow cytometry. Representative flow data (left) and summary plot (right) are shown. **e** CD8 T cells purified from PBMCs or BMMCs were co-cultured with T2 cells (used as antigen presenting cells) pulsed with WT1 or SV40 peptide (used as negative control) for 6 days. After the co-culture, flow cytometry analysis of the intracellular expression of IFN-γ and TNF-α in CD8 T cells was performed (*n* = 4). Representative flow data (left) and statistic summary plot (right) are shown. **f** PD-1 expression on leukemia-reactive CD8 T cells (gated on IFN-γ+ cells, *n* = 4). Representative flow data (top) and statistic plot (bottom) are shown. *P* values were obtained by the paired *t* test

To evaluate the correlation between the PD-1 expression and clinical outcome, we defined high-PD-1 vs. low-PD-1 subgroups in the cohort of AML patients. The mean value of PD-1 expression on CD8 T cells in bone marrow of the 22 AML patients evaluated in our study was used as the cutoff here. Upon analyzing the rate of complete remission (CR) after induction treatment and the overall survival, we found no significant difference between the high-PD-1 and low-PD-1 subgroups (Supplementary Fig. 1). The small sample size in our study precludes a definite conclusion, further analysis with large cohort of patients is warranted to investigate this important question.

In summary, our study demonstrates a significant difference in the T cell immune response between bone marrow and peripheral blood in AML patients. An increased proportion of CD8 T cells within bone marrow expresses PD-1 and these T cells exhibit reduced anti-leukemia response. To our knowledge, this is the first study characterizing the phenotypic signature and anti-leukemia activity of CD8 T cells within bone marrow in AML patients. Our data highlight the importance of evaluating bone marrow specimens when defining the immune status in AML. Importantly our study demonstrates suppressive immune features in bone marrow and provides crucial information for clinical translation of immunotherapy for this devastating disease.

## Electronic supplementary material

Supplemental Table 1(DOCX 13 kb)

Supplemental Figure 1(PPTX 76 kb)
