# Supplementary material for: Bone marrow CD8 T cells express high frequency of PD-1 and exhibit reduced anti-leukemia response in newly diagnosed AML patients
Source: Blood Cancer J. 2018 Mar 21;8(3):34. doi: 10.1038/s41408-018-0069-4 (PMC5862839; doi:10.1038/s41408-018-0069-4)
Supplement: Supplementary file 1 — Supplemental Table 1(DOCX 13 kb) [file 41408_2018_69_MOESM1_ESM.docx]

**Table1. Patient characteristics**

| **Total**  (n=22) |  |
| --- | --- |
| **Age, y**  Median  Range | 57  22-83 |
| **Gender**  Male  Female | 12  10 |
| **WBC, ×10^9^/L**  Mean  Range | 69.8  1.2-199.8 |
| **PB blasts (%)**  Mean  Range | 50.1  1-98 |
| **Absolute blasts count, ×10^9^/L**  Mean  Range | 47.3  0.02-193.8 |
| **BM blasts (%)**  Mean  Range | 62.7  18-97 |
| **Risk Category***  Favorable  Intermediate  Adverse | 2  10  10 |

Abbreviations: WBC, white blood cell; PB, peripheral blood; BM,

bone marrow. *Risk stratification is per 2017 AML European

Leukemia Net (ELN) Recommendations.
