# Supplementary material for: Bone marrow CD8 T cells express high frequency of PD-1 and exhibit reduced anti-leukemia response in newly diagnosed AML patients
Source: Blood Cancer J. 2018 Mar 21;8(3):34. doi: 10.1038/s41408-018-0069-4 (PMC5862839; doi:10.1038/s41408-018-0069-4)
Supplement: Supplementary file 2 — Supplemental Figure 1(PPTX 76 kb) [file 41408_2018_69_MOESM2_ESM.pptx]

## Slide 1
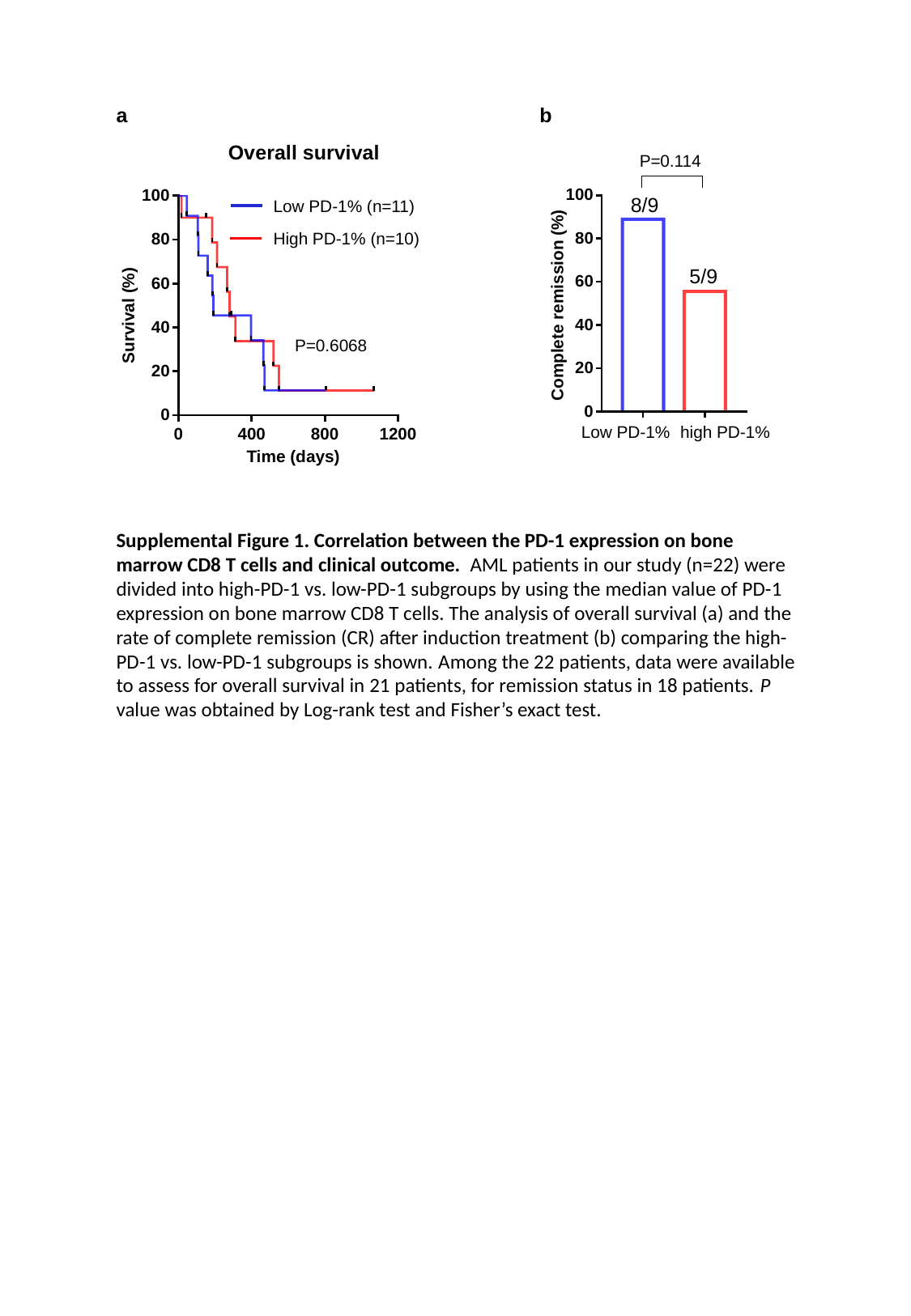

b
a
Overall survival
P=0.114
8/9
Low PD-1% (n=11)
High PD-1% (n=10)
5/9
Complete remission (%)
Survival (%)
P=0.6068
Low PD-1%
high PD-1%
Time (days)
Supplemental Figure 1. Correlation between the PD-1 expression on bone marrow CD8 T cells and clinical outcome. AML patients in our study (n=22) were divided into high-PD-1 vs. low-PD-1 subgroups by using the median value of PD-1 expression on bone marrow CD8 T cells. The analysis of overall survival (a) and the rate of complete remission (CR) after induction treatment (b) comparing the high-PD-1 vs. low-PD-1 subgroups is shown. Among the 22 patients, data were available to assess for overall survival in 21 patients, for remission status in 18 patients. P value was obtained by Log-rank test and Fisher’s exact test.
